# 18 Years of Changing Trends: Swiss Data on the Clinical Characteristics and Game Types Associated with Problem Gambling

**DOI:** 10.3390/healthcare11020166

**Published:** 2023-01-05

**Authors:** Emilien Jeannot, Cheryl Dickson, Coralie Zumwald, Olivier Simon

**Affiliations:** 1 Centre Du Jeu Excessif, Addiction Medicine, Lausanne University Hospital, 1011 Lausanne, Switzerland; 2 Institute of Global Health, Faculty of Medicine, Chemin de Mines 9, 1202 Geneva, Switzerland

**Keywords:** gambling, Switzerland, clinical characteristics

## Abstract

Recent changes in the Swiss gambling context include the introduction of a new gambling policy (Swiss Federal Act on Gambling; GA), and the associated opening of the online gambling market. Within this context it is important to observe evolving trends in gambling behavior. This study therefore aims to identify the gambling behavior and clinical characteristics of a treatment-seeking population over an 18-year period (2001 to 2018). Specifically, the proportion of referrals relating to the three most popular game-types; VLT play, slot machines and online gambling, and associated socio-demographic characteristics, gambling-related spending and clinical features. The results indicate the high addictive potential for VLT and slot-machine use, over the 18-year period with an increasing use of online gambling from 2012 to 2018 (even before the official opening of the market). Problem-gambling vulnerability factors such as low income, unemployment and debts as well as self-reported suicidal ideation, were also over-represented in the sample. This longitudinal overview provides a detailed picture of treatment-seeking referrals, for future comparisons and can be used to tailor clinical interventions.

## 1. Introduction

Opportunities to gamble are widespread and with the rapid expansion of gambling offers, the issue has finally been raised as a global public health concern [1]. Within Switzerland, it is estimated that 71% of the population have gambled in their lifetime and 60% in the past year [2]. Of these, around 3.8% are purported to show gambling-related harm, contributing to an estimated 31.3% of gross gaming revenue [3]. Problem gambling has been defined as a friction or difficulty in any area of functioning that results from some element of gambling behavior [4]. Such difficulties are typically associated with additional clinical and social problems, including anxiety, depression, suicidal ideation, family dysfunction and financial problems [5]. Problem gambling is seen to affect not only individuals who gamble but also close family members such as partners and children [6]. Personal vulnerability factors associated with problem gambling have included ethnicity, gender (male), income (low), age (older) smoking (high consumption), presence of a personality disorder [7]. However, the strongest predictor in this particular study was a preference for electronic games machine [EGM] play. Elsewhere, a correlation has also been found between the density of gambling facilities and levels of problem gambling [8]. This is concerning as Switzerland has a high concentration of gambling facilities. Within French-speaking Switzerland alone there are Some 21 casinos, around 700 video lottery terminals (VLTs), diffuse sports betting and lottery facilities (run through kiosks, cafes, and bars), as well as online gambling [9].

With regard to its historical context, Switzerland has moved from a period of prohibition in the early 1900s to a legally regulated framework that now permits gambling in its diverse forms. In 1998 the Federal Act for Casinos entered into force [10] bringing the requirement for casinos to implement detection and exclusion measures for problematic play. Moreover, in 2005 the cantons implemented an intercantonal convention for lotteries and sports betting [11], which included the introduction of a 0.5% tax on the gross income of the lotteries, to be used for preventative purposes. In January 2019 a new Swiss Federal Act on Gambling (Gambling Act; GA—in French “Loi fédérale sur les jeux d’argent; LJAr”) entered into force, [12], superceding previous legislation and incorporating all forms of gambling including casinos, lotteries, sports betting and online gambling. The GA maintains certain measures such as the 0.5% lottery tax to finance cantonal prevention, and strengthens existing casino detection and exclusion measures whilst extending this requirement to online gambling, offline lotteries and certain sports betting games (those identified as high-risk by the independent regulatory body, Gespa). For an overview of GA see [13]

The new federal act marked the official opening of the Swiss online gambling market. Whilst the impact of this emerging market is yet to be seen, initial studies from other jurisdictions have indicated a link with increased problem gambling [14,15]. In particular, online gambling offers unique features such as constant availability, ease of payment [16] and the opportunity to gamble privately, from home [17], which has been argued by some to make it more addictive than other types of game [18]. Research into the characteristics of people who gamble online have suggested that consumers are more likely to be young males [19] who also participate in other forms of gambling and have a comparatively higher gambling expenditure [20]. A possible overlap with excessive internet use has also been highlighted [19,21].

Another typically available game in Switzerland is slot machines otherwise known as “fruit machines”. These are fixed odds betting terminals, for which the outcome is independently determined, according to the individual machine. Slot machines are argued by some to have become the gambling industry’s revenue mainstay [22] and they are largely available within Swiss casinos. They are inexpensive to run and offer low-stakes betting to a large number of customers, which makes them highly profitable [23]. Enhancements in structural characteristics over recent decades, including near misses, sound, light and color effects and event frequencies are argued to make them increasingly compelling [22,24]. In addition, their multi-line play features are suggested to be particularly absorbing for those with gambling problems [25]. Identified risk factors for disordered gambling include being male, aged 16–25 years, having experienced academic failure and other addictive behaviors [26]. Similarly, a Swiss study found that casino attendees and slot machine players were typically younger (i.e., under 35 years old) than lottery and sports betters, and approximately one third of players were non-Swiss nationals [27].

In addition to slot machines and online gambling, video lottery terminal (VLT) play has also aroused concerns. VLTs are a specific type of electronic games machine (EGM) that is usually available outside of casinos in public venues such as bars, restaurants, and amusement arcades. VLTs function through a central server and so the outcome of games (wins and losses) is determined remotely. They are most often associated with gambling-related problems [28] and have structural characteristics that are deemed to increase playing behavior including near wins (Côté et al., 2003), rapid play [29,30] and use of sounds [31]. Other characteristics, such as the inclusion of a stop button are reported to create the illusion of control, which has been linked to erroneous cognitions [32]. Geospatial studies focusing specifically on VLTs have shown that outlets are often concentrated around socially disadvantaged populations who are at a greater risk of developing gambling problems [33,34] and an initial report indicates that this is also true within Switzerland [9]. This report also found that VLTs accounted for between one third and one half of referrals to a specialized gambling service between 2001 to 2018.

Given the evolving landscape within Switzerland’s gambling domain, including the increasing availability of high-risk games, official opening of the online gambling market and introduction of the new Federal Act, it is particularly important to understand the subsequent impact upon problematic gambling behavior. Longitudinal research carried out in different jurisdictions has demonstrated changing trends in gambling over time; for example, a recent study in the UK has shown an increase in the use of fixed odds betting terminals, alongside poker and sports betting, and a corresponding decreased use of horse and dog-racing and the National Lottery [35]. Similarly, research into other jurisdictions has highlighted a substantial increase in online gambling [18,36]. It will be particularly important to understand transitions for the Swiss population in order to enable a suitable public health response. The present study thus aims to observe the nature of referrals received at a gambling addiction treatment center, over a period of 18 years. This gives an overview of gambling trends, within a treatment-seeking population, prior to implementing the New Swiss Federal Act on Gambling. The insights gained will provide a useful barometer against which to measure future changes (post GA).

In addition to observing gambling trends, it is important to recognize the individual characteristics typically associated with different games. One Swiss study into the sub-population of adolescent problem gambling has identified that young problem gamblers are, amongst other factors, more likely to be male, apprentices, and non-Swiss nationals, and are more likely to smoke cannabis or use alcohol problematically [21]. It is important to develop a similar profile for the adult gambling population, which would complement an understanding of the current gambling market. The present study therefore identifies the sociodemographic characteristics, gambling-related spending and clinical features of people preferring 3 different game-types. This clinical picture can be used to inform recommendations for service development and future monitoring.

### Aims of This Study

The aims of this study are therefore to:

1. Compare trends in the use of three specific game-types (VLTs, slot machines, online gambling) over an 18-year period, for a treatment-seeking population.
2. Observe the sociodemographic characteristics, gambling-related spending and clinical features associated with each game type, for a treatment-seeking population.

## 2. Method

### 2.1. Setting

All patients included in this study were seen at the Center for Excessive Gambling (CJE) at Lausanne University Hospital (CHUV), Switzerland. The CJE is an integrated care center within the Department of Psychiatry’s Service of Addiction Medicine. It serves as a reference center within Switzerland, advocating a public health approach for gambling and electronic media-related disorders. The service offers support and information to frontline professionals, affected individuals and their entourage. Thus, the main focus of the CJE remains gambling, but certain activities also concern behavioral addictions in a broader sense (in particular problems related to the Internet and screen use). Clinically, the services are aimed at people with gambling problems as well as people with other non-substance addictive behaviors (notably related to video games and the Internet, shopping, sexuality). The center also provides counselling for the relatives of those who gamble. Anyone contacting the service (player or close family member) will receive telephone contact from a clinician within the following 24 h, during which a consultation appointment at the CJE is proposed [37]. Given the Swiss context, basic health insurance should cover the cost of the initial appointment, but excess costs can be high (between 300 and 2500 Swiss Francs, equivalent to 320–2700 US Dollars), which can present a barrier to uptake. To avoid this problem, the cost of first consultations can be covered by the CJE [38]

### 2.2. Participants

The population studied includes all new referrals (these are often self-referrals) received by the CJE for gambling-related problems. The data was collected between 2001 (when the CJE was established) and 2018 (the year before the GA came into force). Data was obtained from an anonymized database that is compiled every year, as part of the CJE’s clinical activity report.

Participants were included in the study if they were adults (aged over 18 years), who were recorded in the database as a new case, presenting with gambling-related problems between 2001 and 2018. New cases who had already made a request for help in the past (reopened cases) were not included in the database.

Among the new cases, a significant proportion reported difficulties related to several game-types. A brief overview of these generic cases is presented, to provide a context for the reader. In order to easily describe the difficulties associated with different game-types, we created distinct categories: referrals related to problematic VLT play (VLT play alone, or amongst other games); slot machine play (alone or amongst other games) and online game playing (alone or amongst other games). Other possible game types (for a minority of cases) were problems related to scratch cards, lotos, horse racing, roulette, illegal slot machines, table games (blackjack) and other games (numerical lottery, gambling with stocks and shares).

Following this, in order to avoid obvious methodological biases, our main analysis includes only new cases reporting problem gambling that was related to a single game type. We therefore identified 3 groups: new cases related only to electronic lotteries (VLTs); those related only to slot machines (provided in a land-based casino context), and cases related only to online games (regardless of their type). These categories were selected as they represent the gambling offers most often found among the new requests.

### 2.3. Data Collection

The CJE’s clinical activity database is compiled using the medical records of new referrals. Following referral, individuals undertake one to four clinical interviews, lasting around one hour each. During this initial assessment phase, a clinician explores different aspects of the individual’s background; personal, family and psychiatric history, social and financial situation, current gambling behavior, motivations to play, crisis-related factors, consequences of behavior and needs relating to follow-up. This interview draws upon the motivational approach [39], and has the dual aims of collecting information and supporting motivation to change and commitment to care. The information reported by individuals is documented in medical records. Clinicians then use the medical records to complete the database, which is completely anonymized.

### 2.4. Primary Outcome

New referrals for problem gambling were categorized into several subtypes, based on the type of games the individual reported playing.

3 categories were defined:

#### 2.4.1. VLT Play Only

This category includes new referrals that reported problem gambling in relation to VLT play only. People reporting problems with VLTs in addition to other gambling were not included.

#### 2.4.2. Slot Machines Only

Similarly, this category relates to new referrals reporting problem gambling on slot machines only (which takes place in land-based casinos in Switzerland). People who reported problematic slot machine play alongside problems related to other gambling offers were not included.

#### 2.4.3. Online Gambling Only

This category includes people who reported difficulties with online gambling, (regardless of actual game type). This includes online sports betting, casino games (including slot machines), poker or any other online gambling offer. As with the previous categories, people who reported problems with online gambling alongside land-based betting were not included.

Socio-economic variables were identified (gender, age, marital status, number of children, nationality, education, social insurance, employment) and reported for each of the three groups detailed above. We also analyzed differences in the amounts of self-reported debt and monthly income, for each of the defined categories.

The severity of gambling problems was assessed by the number of diagnostic criteria that were met for pathological gambling disorder (out of a total of 10 criteria in accordance with the old DSM-IV nomenclature) and by the presence or absence of suicidal ideation.

### 2.5. Statistical Analysis

The data was grouped into three, six-year increments to enable three comparable periods, as follows: 2001–2006, 2007–2012, and 2013–2018.

Missing or miscoded data was removed from the analyses. Continuous data was analyzed using means and medians with their confidence intervals; categorical data was analyzed as percentages.

Descriptive statistics were used to present an overview of the data. In addition, categorical variables were compared with chi-square and Fischer tests. Means and medians were compared by Student’s *t* tests and Anova tests. Analyses were performed with Stata14. A *p* of less than 0.05 was chosen as the significance level.

### 2.6. Ethical Aspects

As the health-related data were collected anonymously, the data does not fall under the Swiss Federal Law on Human Research.

The database is stored in a protected file on the unit’s secure server; it does not include any nominative data. Each new case is identified by a code that ensures anonymity. In particular, there is no mention of date of entry or date of birth, as these data are aggregated by period.

Only the clinicians who enter the data have access to the secure server on which the database is stored. It was exported in stata format for analysis by the researcher overseeing data analysis, and did not leave the unit’s internal secure server. All raw data and results are therefore only available on the above-mentioned secure server.

## 3. Results

During the period from 2001–2018, the CJE received 657 new referrals relating to a gambling problem. This represents between 16 and 69 new people being referred per year. The number of requests varies greatly from year to year, with an average of 37 new gambling-related consultations per year. Amongst these new referrals, 27 referrals reported problems relating to scratch cards, lotos, horse racing, illegal slot machines, roulette, table games (black jack) and other games (numerical lottery, gambling on stocks and shares). These individuals did not report playing VLTs, slot machines or online gambling, and so were not included in the sample. A total of 630 referrals were therefore found to report problematic VLT play, slot machine play or online gambling (either alone or amongst other games) and were therefore included in the first part of the study. If we consider the three distinct categories of games we have chosen (VLT play, slot machine play or online gambling only; i.e., without other types of play), a total of 341 referrals were analyzed in the main part of this study.

**Figure 1:**
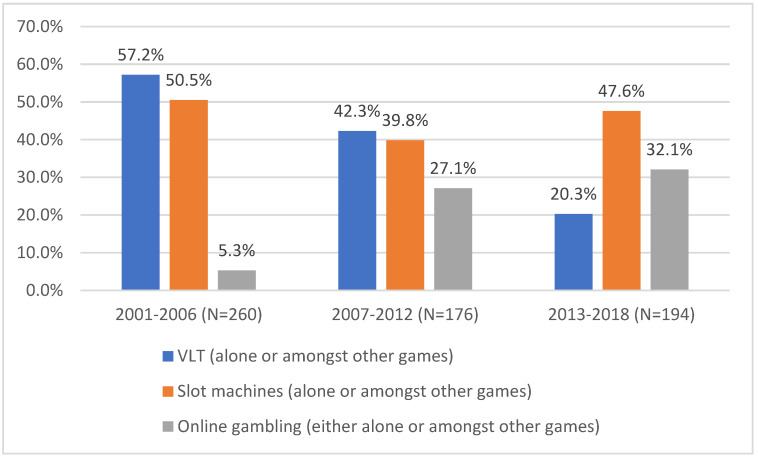
Evolution of referrals between 2001 and 2018 for patients playing VLT, slot machines or online gambling (either alone or amongst other games) (*n* = 630). (Each new referral can play more than one game-type and be represented more than once in the figures, therefore the totals for each time period can exceed 100%.).

Figure 1 presents individuals referred to the CJE (*n* = 630) between 2001 and 2018, for problems related to VLT play, slot machine use or online gambling (either alone or amongst other games). The findings indicate that referrals relating to VLT play (VLT play only or among other games) decrease over time, while that of online gambling (alone or among other games) has been increasing since 2001. The number of people consulting for problematic slot machine use (alone or amongst other games) remains relatively constant during the periods studied.

### Subgroup Comparisons

**Figure 2:**
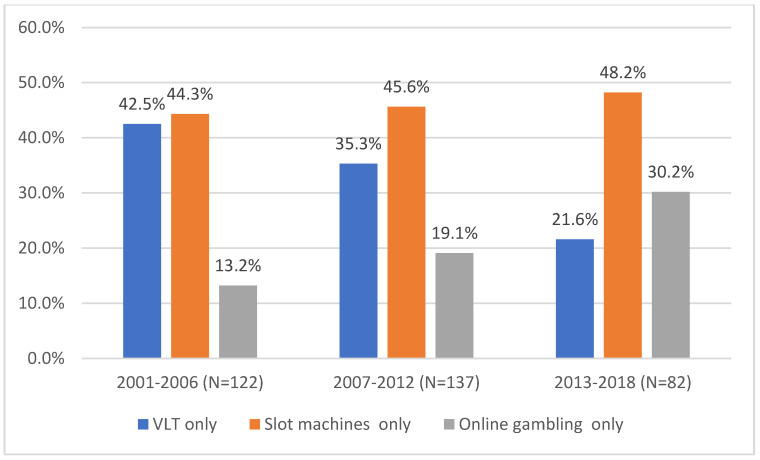
Evolution of referrals between 2001 and 2018 for problems relating to a single game-type (*n* = 341).

Figure 2 presents new cases at the CJE relating to problem gambling for only a single game-type (i.e., VLT play only, slot-machines only, online gambling only: *n* = 341) The findings show that VLT play was the most popular choice of game amongst new referrals for the periods of 2001–2006 (42.3% of requests) and 2007–2012 (35.3% of requests). However, its popularity can be seen to decrease over time and for the most recent period (2013–2018), VLT play appears in third position (21.6% of requests), just behind slot machines alone (48.2%) and before online gambling alone (30.2%). We can also observe that the percentage of people who play slot machines has increased slightly from 44.3% to 48.2%. In addition, a significant increase in the number of those who play online is observed, from 13.2% in 2001–2006 to 30.2% in 2013–2018.

Table 1 presents socio-demographic characteristics of new referrals relating to gambling problems for VLT play only, compared to slot machines only and online gambling only. With respect to gender, males are significantly overrepresented (77.9%) among all new VLT-related referrals. 66.4% of slot machine players over the entire 18 year-period were men. In comparison, a larger proportion of men reported a preference for online gambling (85.5%). The 31–40 and 41–50 age groups account for the majority of new cases related to single game-type. However, it is noted that this proportion is lower for applications related to VLT play only. On the other hand, a comparison of the mean age of this subgroup with that of slot-machine players alone indicates a significant difference: that 51–60-year-olds are more proportionately represented in the former subgroup, while those over 60 are more numerous in the latter. This distribution by age group is similar for VLT play, online gambling and slot machine play.

The data on marital status shows that about one-third of new VLT-related referrals were for single people, about one-third for those who were married, and the remaining one-third for separated, divorced, or widowed people. There is no significant difference between the subgroup of applications related to VLTs alone and those related to slot-machines only or online gambling only.

The majority of players for all categories (VLTs only, slot machines only or online gambling only) had one child or more (between 70% and 65% of referrals).

With regard to the level of education attained, we note that the majority of new referrals for those playing VLTs only, relate to people with an intermediate level of education (Baccalaureate, or apprenticeship: 52.6%) or a low level (compulsory schooling or less: 40%). The comparison between the subgroup of applications related to VLTs only and slot-machines only shows us a highly significant difference between the two subgroups, with the VLT subgroup reporting significantly lower levels of education. There was a comparable level of education between the online gambling group and slot machine groups.

Regarding the use of social insurance, 42% of the VLT group received unemployment benefits, social assistance, disability benefits, AVS (pension) or other social insurance. A comparison of the VLT subgroup with the slot-machine-only or online gambling subgroups does not reveal a significant difference.

Finally, and consistent with the previous results, one third (33%) of the online gambling group reported being unemployed. The results were comparable between the three subgroups for this variable.

Table 2 indicates that people playing VLTs-only reported an average salary of 3700 Swiss francs (3964 US dollars). The people playing slot machines only and online gambling only reported having a slightly higher income 4452 and 4815, respectively (4770 and 5159 US dollars). The median salary was practically identical to the average salary for the three groups. For comparison, the median salary in Switzerland is 6659 Swiss francs (7135 USD).

We can see also that the slot machine subgroup and online gambling subgroup reported having, on average, much higher debts. The reported values were 76,602 and 79,083 Swiss francs, respectively (82,080 and 84,740 US dollars) compared to the VLT subgroup (18,522 Swiss francs–19,850 US dollars). These differences were found to be statically significant (*p* = 0001 and *p* = 0002).

Table 3 shows that there are no statistically significant differences between the three groups regarding self-reported suicidal ideation or the number of DSM IV criteria for gambling disorder that were met. We can see that 34.4% of VLT players reported suicidal ideation compared to 38% for slot machine players and 45.1% for online players. Our studied population has a high number of DSM IV criteria, indeed 46.7% of VLT players reported between 8 and 10 DSM IV (severe gambling disorder) criteria. This figure rises to 47.4% for slot machine players and 51.2% for online gambling.

## 4. Discussion

This study is the first in Switzerland to focus on the clinical characteristics of patients over such a long period of time (18 years). The approach taken enables documentation of changes in clinical characteristics as well as the type of problematic games used by people seeking support from a specialized care center.

The results of this study show that electronic and online gambling are the most represented game types associated with this Swiss, support-seeking sample. Other international studies also show this evolution of gambling offers towards an increase in electronic and online gaming [40]. Indeed, according to a report published by the European Gaming and Betting Association, online gambling revenue in Europe is estimated to grow by 19% in 2022, to reach a gross gaming revenue of 99.5 billion US dollars. Growth of the online gambling market in Europe is expected to continue. In particular, operators’ online gambling revenues are expected to grow by around 9% per year and reach 41% of total gambling revenue in Europe by 2026; an increase of 26% since 2019 [41]. In addition, a rapid increase in the use of mobile games is reported.

This poses all the more serious public health consequences since VLT and slot machines are the most frequently found games in the first two periods, confirming their high addictive potential as shown by other studies [42,43]. We note that online gambling was already progressing significantly and was increasingly used during the last period (2013–2018). This was observed, even though the online gambling market was not yet open in Switzerland (its official opening was in January 2019). We can therefore assume or fear that this type of game has further progressed since 2019 with the entry into force of the new Swiss legislation, bringing serious consequences for its players. Indeed, several studies show that the prevalence of disordered gambling is higher among people who gamble online compared to those who play offline [44,45,46] and that people who gamble online are three to four times more likely to have gambling problems compared to those who play offline [47].

The reported socio-economic characteristics of our sample show an over-representation of people with modest incomes, those who are non-Swiss, unemployed and/or receiving social support. In addition there were significant levels of self-declared debt for participants in the 3 subgroups, and this was a particularly strong characteristic for VLT players. Studies have shown that low socioeconomic status is a risk factor for developing a gambling disorder. Indeed, these studies have revealed that gambling spending by households with a low level of income represents a proportionately higher percentage of their budget than that of wealthier households [48]. However, it appears that it is the social categories that devote a greater share of their disposable income to gambling who are also the most exposed to its consequences. Socio-economic factors such as unemployment, receipt of social benefits, low income-level and immigrant status tend to increase the damage associated with gambling consumption [49]. A study of the US population [50] showed that the problem gambling rate was highest for the quintile that represents the poorest 20% of households (7.5%), while the lowest rate was in the quintile for highest-income households (1.7%).

The results also show that our population of treatment-seeking players present with a profile of severe problems, with the majority showing 8 or more diagnostic criteria out of a maximum of 10, a finding that was consistent for all game types. This result is in-keeping with the international literature, which shows that just over one-third (37.6%) of problem land-based gamblers, benefit from treatment at land-based services and that this figure is even lower (23.5%) for those who gamble online [51]. Moreover, on average, the disorder evolves over the preceding 5 to 6 years before entering into treatment [52]. It therefore appears that the people with less severe disorder profiles are less likely to enter into treatment, and their disorder may therefore worsen. The other concerning result is that more than one third of the sample reported suicidal ideation at the time they entered into treatment, regardless of the type of gambling reported. Suicide risk is a serious concern associated with problem gambling [53]. Particularly, research shows that between 36% to 50% of those seeking treatment for gambling problems have a history of suicidal ideation [54]. Similarly, Mortality and suicide rates are significantly elevated for people who problem gamble, with one study reporting a 15-fold increase in suicide mortality for those with a gambling disorder, when compared to the general population [55].

### 4.1. Strengths of the Study

One of the strengths of this study is that it is based on the observation of a clinical population over 3 time periods, covering a total span of 18 years. Giving an overview of clinical characteristics and game types before the GA entered into force, it provides an important baseline for subsequent post-GA observations. In addition, the relatively large sample size for this type of population in Switzerland allowed us to identify the clinical characteristics according to each specific type of game, showing the commonalities but also some particularities; a clinical picture that can help to better target specific interventions.

At the CJE, the treatment pathway gambling disorder and related concerns is typically multimodal and multifocal, being based on an ad hoc combination of targeted social support, feedback and psychoeducational advice. Psychoeducational interventions are inspired by the Brief Intervention model and, if necessary, medium-term follow-up from a cognitive-behavioral orientation. In cases of co-morbidity, a psychiatric assessment may also take place with one of the two psychiatrists and lead to an integrated follow-up including pharmacotherapy. In all situations, it is proposed to involve relatives [56] and to adjust the networking and subsidiarity setting of the other stakeholders involved. The findings from the present study highlight the importance of prioritizing the social component of the support given. Specifically, the demographic profile of service-users should always be taken into account and culturally sensitive psychoeducational material should be made available in the most common mother-tongues. This issue should also be highlighted when training therapeutic staff, and connections should be made not only with general addiction consultations but also with specialized services for vulnerable groups, especially those with a migrant background.

With regard to the high levels of suicide risk observed, the CJE team is duly trained to provide clinical interventions in times of suicidal crisis [57]. It is also important to maintain close links between this type of consultation work and the health services that receive people in the context of medical emergencies, including psychiatric liaison consultation services of general hospitals.

### 4.2. Limitations of the Study

This study includes a treatment-seeking population of people with gambling-problems, and therefore a small minority of people affected by gambling. This is particularly true as few problem gamblers are reported to seek professional help [20]. Moreover, the present study includes data from only one support service (the CJE) in French-speaking Switzerland and is not necessarily representative of people who consult services across the whole of Switzerland.

As the salary and debts are self-reported by those consulting, they may be affected by a perceptual bias or subject to false declaration. Moreover, since the presence or absence of DSM IV criteria for gambling disorder were determined by the interviewing clinician, reports may vary from one professional to another. Fluctuations in the number of criteria for the same person have been seen to exist, which suggests that this information should be considered as a general overview rather than a precise measure. Standardization of data collection methods should make it possible to limit inter-clinician fluctuations, in future studies.

Another limitation within this study is that the main reason for consultation (most problematic game, according to the person) is not indicated within the clinical database. In other words, all gambling problems regardless of the category of games are put at the same level. It would be useful to identify the main reason for consultation, in subsequent studies. An adjustment to data collection processes could, again, be undertaken to make this possible. In addition, to better understand the data, we excluded people who came to consult with several different gaming problems (multi-players). This removed a significant number of participants from the study and is likely to have included the most problematic players, with the highest levels of spending and debts.

## 5. Conclusions

As a result of this study, we can ensure that clinical practice remains sensitive to the needs of the presenting population. In addition, we can recommend better documentation of characteristics and monitoring to bring support to these players. In line with the new legislation, we should look at the characteristics of players to understand who is at risk. The impact of the newly opened online gambling market will need to be observed, and the present data can provide a valuable baseline for comparison. Within the context of the new regulatory framework, which stipulates the need to designate resources to research, it is essential to allocate ad hoc funds towards future research and monitoring efforts. Such research will need to be incorporated into a systematic and forward-looking framework, taking into account the ethical constraints linked to the creation of databases relating to consulting populations. Going forwards, it will be particularly important to observe any changes in presenting cases and adapt clinical work and available support services in line with future trends.

## Figures and Tables

**Table 1:** Sociodemographic characteristics of new referrals by subtype.

	VLTs Only *n* = 122		Slot Machines Only *n* = 137		Online Gambling Only *n* = 82		
**Gender**	*n*	%	*n*	%	*n*	%	p *
Female	27	22.1%	46	33.6%	12	14.5%	**0.035**
Male	95	77.9%	91	66.4%	70	85.5%	
**Age range**							
18–20	0	0.0%	1	0.7%	1	1.2%	0.07
21–30	18	14.8%	19	13.9%	7	8.5%	
31–40	25	20.5%	36	26.3%	24	29.3%	
41–50	33	27.0%	39	28.5%	22	26.8%	
51–60	42	34.4%	24	17.5%	15	18.3%	
61–70	4	3.3%	18	13.1%	13	15.9%	
**Civil status**							
Single	28	23.0%	38	27.7%	24	29.3%	0.06
Married	58	47.5%	53	38.7%	32	39.0%	
Separated	14	11.5%	12	8.8%	7	8.5%	
Divorced	20	16.4%	30	21.9%	15	18.3%	
Widowed	2	1.6%	4	2.9%	4	4.9%	
**Parental status**							
No children	36	29.5%	42	30.9%	28	34.1%	0.3
1 or more children	86	70.5%	94	69.1%	54	65.9%	
**Nationality**							
Swiss	52	42.6%	73	53.3%	45	54.9%	0.08
Other (European, African…)	70	57.4%	64	46.7%	37	45.1%	
**Education**							
University, higher education	10	8.2%	25	18.2%	18	22.0%	**0.025**
Baccalaureate, apprenticeship	63	51.6%	72	52.6%	40	48.8%	
Compulsory schooling/Uncompleted compulsory schooling	49	40.2%	40	29.2%	24	29.3%	
**Social support**							
No income support	76	62.3%	80	58.4%	52	63.4%	0.6
Disability benefit	10	8.2%	13	9.5%	5	6.1%	
Pension	5	4.1%	16	11.7%	10	12.2%	
Social assistance	8	6.6%	7	5.1%	5	6.1%	
Unemployment benefit	18	14.8%	17	12.4%	8	9.8%	
Other (e.g., back to work supplement...)	5	4.1%	4	2.9%	2	2.4%	
**Employment**							
No	37	30.3%	38	27.7%	27	32.9%	0.09
Yes	78	63.9%	85	62.0%	47	57.3%	
Retired	5	4.1%	11	8.0%	6	7.3%	
Other	2	1.6%	3	2.2%	2	2.4%	
**Gambling debts**							
No	31	25.4%	35	25.5%	23	28.0%	0.8
Yes	91	74.6%	102	74.5%	59	72.0%	

* Statistically significant differences are shown in bold in the table (*p* less than 0.05).

**Table 2:** Debts and income according to player-type.

Average Income Reported by Month (Swiss Francs)	VLT Only *n* = 32		Slot Machines only N = 41		Online Gambling Only *n* = 24		p *
Moyenne IC 95%	3700	3298–4101	4452	3924–4980	4815	4025–5606	0.07
Median	3850	-	4200	-	4800		0.2
**Gambling debts**							
Moyenne IC 95%	18,522	13,078–23,966	76,602	40,976–11,2228	79,083	32,387–125,779	**0.001**
Median	5000	-	25,000	-	42,500	-	**0.002**

* Statistically significant differences are shown in bold in the table (*p* less than 0.05).

**Table 3:** Suicidal ideation and DSM IV criteria by player type.

.	VLT Only *n* = 122		Slot Machines Only *n* = 137		Online Gambling Only *n* = 82		
**Suicidal ideation (SI)**	N	%	N	%	N	%	*p*
No SI	80	65.6%	85	62.0%	45	54.9%	0.2
SI	42	34.4%	52	38.0%	37	45.1%	
**Number of DSM criteria**							
Less than 3 criteria (non-problematic)	5	4.1%	6	4.4%	3	3.7%	0.9
3 to 4 (problematic)	20	16.4%	23	16.8%	15	18.3%	
5 to 7 (gambling disorder)	40	32.8%	43	31.4%	22	26.8%	
8 to 10 (severe gambling disorder)	57	46.7%	65	47.4%	42	51.2%	

## Data Availability

Data will be made available by the authors upon reasonable request.
